# Prehospital use of pelvic circumferential compression devices in a physician-based emergency medical service: A 6-year retrospective cohort study

**DOI:** 10.1038/s41598-020-62027-6

**Published:** 2020-03-20

**Authors:** Tobias Zingg, Romain Piaget-Rossel, Julie Steppacher, Pierre-Nicolas Carron, Fabrice Dami, Olivier Borens, Roland Albrecht, Vincent Darioli, Patrick Taffé, Ludovic Maudet, Mathieu Pasquier

**Affiliations:** 1 Department of Visceral Surgery, Lausanne University Hospital - CHUV, Lausanne, Switzerland; 2 Center for Primary Care and Public Health, University of Lausanne, Lausanne, Switzerland; 3 School of Medicine and Biology, University of Lausanne, Lausanne, Switzerland; 4 Department of Emergency Medicine, Lausanne University Hospital - CHUV, Lausanne, Switzerland; 5 Department of Orthopedics and Traumatology, Lausanne University Hospital - CHUV, Lausanne, Switzerland; 6 Swiss Air Rescue REGA, Zurich, Switzerland

**Keywords:** Bone, Trauma

## Abstract

Fractures of the pelvic ring are a potential source of significant bleeding. Pelvic circumferential compression devices (PCCDs) can reduce and immobilize unstable fractures, but their hemostatic effect is unproven. Our aim was to assess the current practice of prehospital PCCD application and to identify factors available in the field predictive of significant pelvic ring injuries. All interventions (n = 13,435) in the Lausanne University Hospital Emergency Medical Service (EMS) were screened for PCCD placements from January 2008 to November 2014. Significant pelvic ring injuries (Tile types B or C) were considered as potentially benefitting from a PCCD. Data were extracted from the local prehospital registry. During the study period, 2366 trauma missions were performed. A PCCD was applied to 552/2366 (23%) patients. Significant pelvic ring injuries were present in 105/2366 (4.4%). Factors associated with the presence of significant pelvic ring injury were increased respiratory rate (OR 1.04), prolonged capillary refill time (OR 2.11), increased shock index (OR 3.91), pedestrians hit by a vehicle (OR 2.19), and presenting with falls from more than 2 m (OR 1.91). Among patients with a significant pelvic ring injury, a PCCD was placed in 79 (75%) and omitted in 26 (25%). One sixth of patients with a PCCD had a final diagnosis of significant pelvic ring injury. Further studies are needed to better understand which patient-, or accident-related factors are associated with prehospital PCCD omission among patients with significant pelvic ring injury.

## Introduction

Trauma associated with pelvic fractures carries a high morbidity and mortality^1,2^. Disruption of the pelvic ring not only acts as a marker for the high amount of kinetic energy absorbed by the body at the time of impact but may also cause significant blood loss into the retroperitoneal space from fractured bone surfaces, disruption of the pelvic venous plexus, and/or torn branches of the internal iliac arteries^3,4^. Active arterial bleeding can be managed in the hospital either non-surgically with arterial angioembolization^5^ or surgically with preperitoneal pelvic packing^6^, alone or in combination with arterial angioembolization^7,8^. While several studies have shown the effectiveness of PCCDs in terms of fracture reduction^9–11^, data on their hemodynamic efficiency are lacking or controversial^12–14^. There have also been concerns for masking the severity^15^ and for potentially worsening certain types of injury by PCCD application^11,16^.

Fractures that potentially benefit from a PCCD are pelvic ring injuries which are rotationally (Tile type B) or vertically (Tile type C) unstable^17,18^. Readily applicable, PCCDs may be used in the prehospital setting with the aim of limiting blood loss from pelvic ring injuries as early as possible^19,20^. Although this practice is widely accepted and recommended^21–24^, specific data on prehospital PCCD application have not been available until recently^13,25,26^, and no consensus exists regarding the indications for prehospital PCCD application.

The primary aim of the present study was to assess the current practice in terms of prehospital PCCD application in a physician-staffed EMS. Secondary aims were to identify prehospital predictors of significant pelvic ring injury and those associated with the application or omission of PCCDs among these patients.

## Methods

### Design

Retrospective cohort study based on data from the prospectively collected pre-hospital database of the Emergency Department of Lausanne University Hospital (CHUV), Switzerland.

### Setting

Pre-hospital rescue is performed by physicians with training in emergency medicine intervening through either a ground-based emergency resuscitation vehicle or a rescue helicopter (Swiss Air Ambulance - Rega). Since 2006, PCCD application has been recommended at Lausanne University Hospital EMS for all patients with a potential pelvic injury having sustained a high-energy trauma and/or presenting with hemodynamic instability without an obvious etiology. The commercial PCCDs used in the present series were the SAM Pelvic Sling II^TM^ (SAM Medical Products, Oregon, USA) for the ground-based EMS and the T-POD^TM^ (Cybertech Medical, California, USA) for the helicopter-based EMS. Mission data were collected by the physician in charge of the patient. The collected data included pre-hospital items as well as information on the initial 48-h in-hospital period.

### Materials

We screened all EMS missions from January 2008 to November 2014. We excluded non-traumatic cases, secondary missions (interhospital transfers), and patients dead on scene. We extracted the following data from the database: injury mechanism, age and gender of the patient, vital parameters in the field and at hospital admission, and field management (including equipment with a PCCD). Capillary refill time is defined in our setting as the time required for skin to return to its original colour after blanching it by finger compression^27^. A time longer than two seconds was considered as prolonged. We defined the shock index (SI)^28^ as heart rate/systolic blood pressure. The prehospital acuity of the case was graded using the NACA (National Advisory Committee for Aeronautics) score^29^, which is attributed by the pre-hospital physician at the end of each rescue mission. The available outcomes from the initial 48-h in-hospital period were diagnosis, injury severity scale score (ISS)^30^, and vital status.

For patients with a 48-h diagnosis of pelvic injury or those who received a PCCD in the field, we extracted data on the entire hospital stay from the electronic patient record. One of the authors (JS) confirmed the presence or absence of a pelvic ring injury through analysis of the medical chart (discharge letter, radiological images and reports). We classified each pelvic fracture into one of the 3 main categories (A, B, or C) according to the modified Tile AO classification^31^ by one of the authors (JS). Fractures for which a doubt subsisted were further cross-checked by a senior trauma surgeon (OB). We defined significant pelvic ring injuries as Tile B or C pelvic fractures, since these potentially benefit from a prehospital PCCD. Tile A fractures and isolated fractures of the acetabulum were not considered as potentially benefitting from a PCCD. Presence of a femoral fracture or hip dislocation was also registered.

### Outcome measures

The primary outcome measure was the performance assessment of current practice in terms of prehospital PCCD application. We considered using a PCCD when a Tile type B or C fracture was present and not using a PCCD in the absence of these fracture types on final diagnosis as optimal performance. Secondary outcomes were identification of factors available in the field predictive of the presence of a significant pelvic ring injury and identification of factors associated with the application or omission of PCCD placement in patients with an unstable fracture pattern.

### Statistical analysis

We first compared patients with Tile B/C fractures to patients without fractures according to initial vital signs and trauma characteristics. The statistical significance of the differences was assessed using a two-sample *t*-test for continuous variables and Pearson’s χ^2^ test for discrete ones. Then, we constructed a predictive logistic regression model for Tile B/C fractures based only on prehospital factors. To avoid the issue of overfitting, we only included seven variables into our predictive model^32^. These variables were selected according to their clinical relevance. In the final model, we retained three continuous variables: age, respiratory rate at the scene, shock index (SI) at the scene; three binary variables: one indicator for male patients, one indicator for capillary refill time of more than 2 seconds at the scene, one indicator for a GCS (Glasgow Coma Scale) lower than or equal to 8 at the scene; and one categorical variable: trauma characteristics (4 wheels, 2 wheels, pedestrian hit, fall> 2 meters, other). The GCS variable was dichotomized to reduce the number of parameters in the model. The goodness-of-fit of the model was evaluated according to the Hosmer-Lemeshow test and a residuals analysis, both concluding of a satisfactory fit. Nagelkerke’s^33^ pseudo-R^2^ of our regression model was 0.144. We used fractional polynomial methods to search for better specifications than the linear one and found no improvement when using smoother specifications. The issue of missing values was tackled using sequential imputations based on chained equations^34^. Finally, among patients suffering a Tile B/C fracture, we analyzed prehospital factors associated with PCCD application and contrasted PCCD with no-PCCD patients according to the treatment they received, their evolution, and outcomes. Here also, statistical significance was tested using either a 2-sample *t*-test or Pearson’s χ^2^ test. All statistical analyses were conducted using Stata version 14.2 (College Station, Texas, USA).

### Research Ethics

The study was approved by the Human Research Ethics Committee of the Canton of Vaud (CER-VD; protocol number 118/15). The need for informed consent was waived and all methods were performed according to the relevant guidelines and regulations. The manuscript was structured according to the STROBE guidelines for reporting observational studies^35^.

### Ethical approval

This study was approved by the Human Research Ethics Committee (CER-VD; protocol number 118/15). The need for informed consent was waived and all methods were performed according to the relevant guidelines and regulations.

## Results

**Figure 1:**
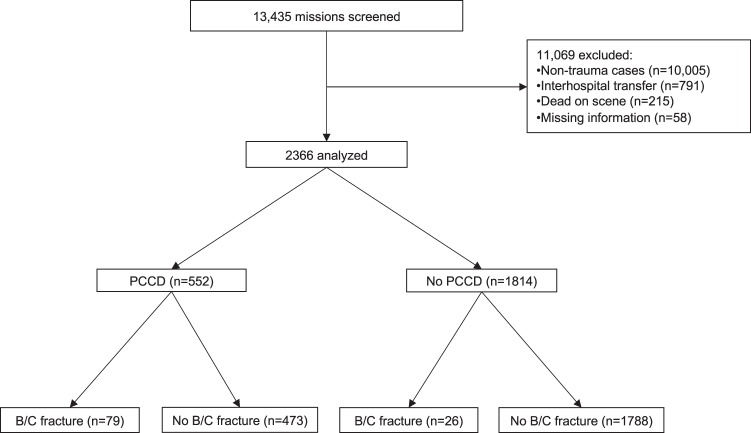
*Flow diagram of the study patients*. *PCCD: Pelvic Circumferential Compression Device; B/C: Tile type B/Tile type C*.

Among the 13,435 missions that occurred during the study period, 2366 patients met the inclusion criteria. PCCDs were applied in the prehospital setting to 552 (23%) patients. A significant (Tile B or C) pelvic ring injury was identified in 105 (4.4%) patients (Fig. 1).

Out of the 552 patients on which a PCCD was applied in the field, 79 (14%) had a significant pelvic ring injury. Among the 473 (86%) patients without significant pelvic ring injury but having a PCCD applied in the field, 179 (38%) suffered from another pelvic or femoral fracture or dislocation (Table 1). A PCCD was not placed in 26 out of the 105 patients (25%) with significant pelvic ring injuries. The characteristics of the study population, overall and with regard to the presence or absence of significant pelvic ring injury, are summarized in Table 2.

**Table 1:** Pelvic circumferential compression device (PCCD) placement according to the type of pelvic and femoral trauma.

	Total n = 2366	PCCD placed^c^ n = 552 (23)
**Significant pelvic fracture, n (%)**	**105**	**79 (75)**
Tile B1	20	17 (85)
Tile B2	58	40 (69)
Tile B3	4	3 (75)
Tile C	23	19 (83)
**No significant pelvic fracture, n (%)**	**2261**	**473 (21)**
**Proximity injuries, n (%)**	**338**	**179 (53)**
Femoral fracture	218^a^	103 (47)
Tile A fracture	65	39 (60)
Acetabular fracture	34	25 (74)
Hip dislocation	21^b^	12 (57)

^a^Among these, 25 also had a significant pelvic fracture, 23 of the latter having a PCCD placed.

^b^Among these, 2 also had a significant pelvic fracture, both having a PCCD placed.

^c^For patients with a PCCD placed in the field, the time between arrival of the EMS physician and hospital arrival was of 36 ± 16 minutes.

**Table 2:** Patient and trauma characteristics according to pelvic ring fracture status.

	Total n = 2366	Tile B/C fracture n = 105	No Tile B/C fracture n = 2261	*P* value
**Patient characteristics**				
Age, mean (SD)	47 (21)	44 (19)	47 (21)	0.15
Male, n (%)	1586 (67)	67 (64)	1519 (67)	0.47
NRS^b^ scale, mean (SD) (n = 1519)	5.4 (3.4)	6.8 (2.6)	5.3 (3.4)	0.0007
NACA^d^ score, n (%)	3.7 (0.95)	4.4 (0.77)	3.7 (0.94)	<0.0001
<4	1070 (45.2)	11 (10.5)	1059 (46.8)	<0.0001
4	833 (35.2)	48 (45.7)	785 (34.7)	0.02
5	389 (16.5)	39 (37.1)	350 (15.5)	<0.0001
6	74 (3.1)	7 (6.7)	67 (3.0)	0.033
**Initial vital signs**				
Respiratory rate, min^−1^ (SD) (n = 2274)	18 (6)	20 (8)	18 (6)	0.0001
S_p_O_2_, % (SD) (n = 2017)	96 (6)	94 (11)	96 (6)	0.005
Heart rate, min^−1^ (SD) (n = 2226)	86 (21)	93(23)	86 (21)	0.002
Systolic blood pressure, mmHg (SD) (n = 2142)	118 (31)	104 (27)	119 (31)	<0.0001
Shock index, mean (SD) (n = 2066)	0.78 (0.27)	0.95 (0.38)	0.77 (0.26)	<0.0001
Shock index>1, n (%) (n = 2066)	293 (14.2)	30 (33.7)	263 (13.3)	<0.0001
Capillary refill time>2 sec (n = 2310), n (%)	216 (9.4)	27 (25.7)	189 (8.6)	<0.0001
GCS^a^, mean (SD) (n = 2360)	13 (3)	12 (4)	13 (3)	0.003
**Trauma characteristics**				
ISS^c^, mean (SD) (n = 2353)	10 (11)	22 (14)	9 (10)	<0.0001
Trauma type				
4 wheels, n (%)	367 (15.6)	23 (23.7)	344 (15.2)	0.02
2 wheels, n (%)	295 (12.5)	14 (14.4)	281 (12.4)	0.56
Pedestrian hit, n (%)	91 (3.9)	12 (12.4)	79 (3.5)	<0.0001
Fall>2 meters, n (%)	315 (13.4)	30 (30.9)	285 (12.6)	<0.0001
Other, n (%)	1287 (54.6)	18 (18.6)	1269 (56.2)	<0.0001

^a^Glasgow Coma Scale.

^b^Numeric Rating Scale, only evaluated for patients with GCS > 13 (n = 1519).

^c^Injury Severity Scale.

^d^National Advisory Committee for Aeronautics.

The results of logistic regression showed that respiratory rate, increased SI, prolonged capillary refill time, pedestrians hit by a vehicle, GCS ≤ 8, and falls from more than 2 m were all variables positively correlated with significant pelvic ring injury (Table 3). All effects were statistically significant except for GCS ≤ 8 (p = 0.42).

**Table 3:** Multivariable analyses of the factors associated with Tile type B or C fractures.

	OR	95% CI	*P* value
**Patient characteristics**			
Age	0.998	0.987–1.009	0.67
Male sex	0.740	0.477–1.148	0.18
**Initial vital signs**			
Respiratory rate	1.036	1.007–1.066	0.01
Shock index^a^	3.912	1.994–7.674	<0.001
Capillary refill time>2 sec	2.109	1.216–3.658	0.01
GCS^b^ ≤8	1.260	0.714–2.225	0.42
**Trauma characteristics**			
4 wheels	ref.	ref.	ref.
2 wheels	0.845	0.418–1.709	0.64
Pedestrian hit	2.192	0.998–4.814	0.04
Fall>2 meters	1.913	1.047–3.497	0.04
Other	0.247	0.128–0.477	<0.001

^a^Shock index = Heart Rate/Systolic Blood Pressure.

^b^Glasgow Coma Scale.

When the analysis was restricted to patients having a significant pelvic ring injury, lower pain levels, low NACA scores (<4), or patients who were hit as pedestrians were more likely not to be equipped with a PCCD (Table 4). Field interventions, evolution, as well as hospital treatments and outcomes of the 105 patients with significant pelvic fractures are presented in Table 5.

**Table 4:** Analysis of prehospital factors associated with pelvic circumferential compression device (PCCD) omission in cases of Tile type B or C pelvic ring fracture.

	Total n = 105	PCCD n = 79	No PCCD n = 26	*P* value
**Patients characteristics**				
Age, years	44 (18.7)	43 (18)	47 (21)	0.40
Male, n (%)	67 (64)	51 (65)	16 (62)	0.79
NRS^b^ scale, mean (SD) (n = 61)	6.8 (2.6)	7.2 (2.3)	5.6 (3.1)	0.03
NACA^c^ score, n (%)	4.4 (0.77)	4.5 (0.68)	4.1 (0.95)	0.03
<4	11 (11)	4 (5.1)	7 (27)	0.002
4	48 (46)	36 (46)	12 (46)	0.96
5	39 (37)	35 (44)	4 (15)	0.01
6	7 (6.7)	4 (5.1)	3 (12)	0.25
**Initial vital signs**				
Respiratory rate, min^−1^ (SD) (n = 102)	20 (8)	21 (8.8)	20 (4.8)	0.57
S_p_O_2_, % (SD) (n = 88)	94 (11)	94 (13)	96 (6)	0.47
Heart rate, min^−1^ (SD) (n = 99)	93 (23)	93 (24)	92 (20)	0.89
Systolic blood pressure, mmHg (SD) (n = 93)	104 (27)	104 (27)	106 (25)	0.81
Shock Index, mean (SD) (n = 89)	0.9 (0.4)	1 (0.4)	0.9 (0.3)	0.77
Shock index>1 (n = 89), n (%)	30 (34)	22 (34)	8 (32)	0.83
Capillary refill time>2 sec, n (%)	27 (26)	24 (30)	3 (12)	0.06
GCS^a^, mean (SD)	12 (4.3)	12 (4.4)	13 (4)	0.49
**Trauma characteristics**	n = 97	n = 71	n = 26	
4 wheels, n (%)	23 (22)	18 (23)	5 (19)	0.53
2 wheels, n (%)	14 (13)	13 (17)	1 (3.8)	0.07
Pedestrian hit, n (%)	12 (11)	4 (5.1)	8 (31)	0.001
Fall>2 m, n (%)	30 (29)	26 (33)	4 (15)	0.045
Other, n (%)	18 (17)	10 (13)	8 (31)	0.06

^a^Glasgow Coma Scale.

^b^Numeric Rating Scale, only evaluated for patients with GCS > 13 (n = 61).

^c^National Advisory Committee for Aeronautics.

**Table 5:** Treatment, evolution, and outcomes of the 105 patients with Tile type B or C pelvic ring fractures and association with pelvic circumferential compression device prehospital placement (PCCD).

	Total n = 105	PCCD n = 79	No PCCD n = 26	*P* value
**Pre-hospital management**				
Cervical collar use (n = 94), n (%)	89 (95)	69 (98)	20 (83)	0.004
Full spinal immobilization (n = 97), n (%)	95 (98)	71 (100)	24 (92)	0.02
Oxygen administration, n (%)	98 (93)	78 (99)	20 (77)	<0.0001
Advanced airways, n (%)	18 (17)	15 (19)	3 (12)	0.38
Peripheral venous access, n (%)	99 (94)	75 (95)	24 (92)	0.62
Intraosseous access, n (%)	2 (1.9)	2 (2.5)	0 (0)	0.41
Intravenous infusion>500 mL, n (%)	41 (39)	38 (48)	3 (12)	0.001
Fentanyl administration, n (%)	60 (57)	46 (58)	14 (54)	0.70
Ketamine administration, n (%)	24 (23)	22 (28)	2 (7.7)	0.03
Vasopressors administration^a^, n (%)	22 (21)	18 (23)	4 (15)	0.42
**First vital parameters at hospital admission**				
Respiratory rate, min^−1^ (SD) (n = 91)	18 (5.7)	19 (10)	18 (5.7)	0.67
Heart rate, min^−1^ (SD) (n = 96)	94 (28)	97 (26)	83 (30)	0.03
S_p_O_2_, % (SD) (n = 87)	95 (9)	95 (10)	98 (3)	0.15
Systolic blood pressure, mmHg (SD) (n = 77)	75 (17)	74 (14)	80 (24)	0.18
Shock Index, mean (SD) (n = 77)	1.3 (0.4)	1.3 (0.4)	1.1 (0.4)	0.07
GCS^b^, mean (SD) (n = 97)	11 (5)	11 (5)	12 (4)	0.23
NACA^c^ score, mean (SD)	4.4 (0.8)	4.5 (0.7)	4.1 (1)	0.03
**Hospital management**				
Emergent hospital intervention <24 h	46 (44)	39 (49)	7 (27)	0.045
External/internal fixation, n (%)	40 (38)	35 (44)	5 (19)	0.02
Angio-embolization, n (%)	4 (4)	2 (2.5)	2 (8)	0.23
Preperitoneal packing, n (%)	2 (2)	2 (2.5)	0 (0)	0.41
**Outcomes**				
ISS^d^, mean (SD) (n = 102)	22 (14)	24 (13)	17 (17)	0.06
48-h mortality, n (%) (n = 97)	20 (21)	15 (21)	5 (19)	0.84
ICU^e^ length of stay, days (SD) (n = 96)	2.5 (5.2)	3.2 (5.9)	0.7 (1.6)	0.03
Hospital length of stay, days (SD) (n = 96)	16 (19)	19 (21)	9 (7)	0.02

^a^Ephedrine (n = 15); epinephrine (n = 7); phenylephrine (n = 3).

^b^Glasgow Coma Scale.

^c^National Advisory Committee for Aeronautics.

^d^Injury Severity Scale.

^e^Intensive Care Unit.

## Discussion

To our knowledge, this is the largest study assessing prehospital PCCD application. PCCDs were applied in the field to 23% of all trauma patients managed by EMS physicians. A significant pelvic ring injury was present in one out of six patients equipped with a PCCD. On the other hand, one out of four patients with a final diagnosis of a significant pelvic ring injury had no PCCD applied.

Several studies have specifically addressed prehospital PCCD application^13,25,26,36^. The majority of data available from the literature originate from in-hospital studies which show a favorable effect of PCCDs for fracture stabilization^10,37–42^, whereas only a few studies suggest a benefit in terms of hemodynamics, bleeding, or mortality^43–45^. In Tile type A fractures, the pelvic ring is intact, and a PCCD has no potential therapeutic impact. Hospital data suggest that PCCDs seem to be most efficient for Tile type B1 (open book) and C fractures, with a less obvious benefit for Tile type B2 fractures (lateral compression)^37^. Type B2 fractures were present in 40 (7%) of our patients with a PCCD. Other studies also recommend PCCD application for B2 fractures, as it did not result in complications or a significant over-reduction of such fractures^37,38^. Despite this controversy, prehospital PCCD application is now widely recommended in case of a suspected pelvic injury^21^.

Reliable clinical identification of the specific type of injury to the pelvic region in the field is difficult. A recent study has shown a sensitivity of 45% and a specificity of 93% for the prehospital clinical exam by an emergency physician for the diagnosis of a pelvic ring injury^25^. In the present series however, there were more patients (n = 179) with a diagnosis of either a Tile A or acetabular fracture, a femur fracture, or a hip dislocation than patients with a significant pelvic ring injury (n = 79) among the 552 with a PCCD applied in the field. Some authors have raised concerns about the liberal use PCCD in the prehospital setting for fear of exacerbating dislocation of other injuries, such as fractures of the acetabulum, proximal femur and iliac wing, thereby causing additional injury and pain^16^. With this assumption in mind, a more specific application policy may be beneficial for the patient. Although subject to documentation bias, we have not observed any complications (pressure ulcers, over-reduction of other injuries or increased pain) among patients with a PCCD placed in the absence of a significant pelvic fracture.

The rate of prehospital PCCD application for Tile B (73%) and Tile C (83%) fractures in our study was higher than those described by two recent studies from the Netherlands^46^ (Tile B: 15%; Tile C: 19%) and from Germany^13^ (Tile B/C combined: 58%). This difference may be explained by the indication for prehospital PCCD application. In the present study period, PCCD use in the field was recommended in the presence of a potential pelvic injury with a high-energy mechanism and/or hemodynamic instability without an obvious cause. However, despite these liberal application criteria, one out of four patients with a definitive diagnosis of Tile B or C pelvic ring fracture had no prehospital PCCD applied. This rate of significant pelvic ring injury in patients without PCCDs applied may be too high, given the potential benefit, the relatively low cost^21^ and low reported complication rates^9^ of PCCD application. One reason for those “missed” injuries may be non-adherence to the local protocol itself, which seems unlikely given the specific setting (university teaching hospital with uniform introduction courses, regular supervision and debriefings). Another reason may be poor identification of situations at risk or population subtypes at higher risk of under-triage (e.g. the elderly). Finally, this may also reflect the limited sensitivity of the protocol itself in the detection of significant pelvic ring injury in the field. Our rate of patients with significant fractures and no binder placed is similar to the 31% described in a recent study^36^ in a setting with also a low threshold for PCCD application in the field. Our higher rate of PCCD application for patients with pelvic ring injury comes with the cost of a higher overtriage rate, as only 14% of patients with PCCDs placed in the field in our study had a significant pelvic fracture, compared to 40% in the study by Yong *et al*.^36^, and 36% in the study by McCreary *et al*.^26^.

In the present series, patients exhibiting signs of shock (increased respiratory rate, capillary refill time>2 seconds, increased SI) were at risk for a significant fracture. As these parameters are indeed well known for their ability to predict severe injury in trauma patients, their sensitivity remains, however, poor^47^. Another risk factor for significant pelvic fracture was the type of trauma. A patient hit by a vehicle or sustaining a fall >2 meters had odds of a Tile B/C fracture 2.19 and 1.91 times larger, respectively, than those of a patient who had a car accident. The mechanism of a pedestrian being hit by a vehicle may be especially relevant, as it was also significantly associated with the fact that a PCCD was not placed on a patient who was later diagnosed with a significant pelvic ring injury. In addition to sensitizing prehospital personnel to the potential benefits of PCCD application, inclusion of these parameters in prehospital PCCD application may help to better identify patients at risk and to maintain this “under-triage” at the lowest possible rate.

Although no significant difference in mortality was observed, patients with PCCDs placed in the field had a longer ICU and total hospital length of stay compared to patients without PCCDs in our study. This most likely reflects the higher degree of injury severity and physiological instability in the first patient group, as patients with PCCDs placed in the field had higher heart rates, oxygen and fluid requirements, as well as higher ISS and NACA scores. However, these results may also reflect the absence of any benefit or even harm from the application of PCCDs. Further studies focusing on potentially detrimental effects of pelvic binders are needed.

The present work has obvious limitations inherent in its retrospective nature. Data accuracy is subject to documentation errors in the registry and patient record. Transfusion requirements and in-hospital duration of PCCD application were not available. The interobserver reliability of the Tile classification system has been previously described as low and insufficient for research purposes^48^. In a previous paper – using a different set of observers - on a cohort with all pelvic ring injuries admitted to our hospital, we obtained a different distribution of fracture types^49^. Although the present study included only patients treated in a physician-staffed prehospital setting, the interobserver variability may influence our results and limit their value.

The potential effect of PCCDs is probably most important for Tile B1 and C fractures^37^, and their efficiency for Tile B2 fractures is debatable^38^. We have included Tile B2 fractures in the types with a potential benefit, since it was shown that B1 fractures may radiologically appear as B2 fractures after PCCD application^15^. It would have been helpful to identify factors associated with the omission of PCCD placement for patients with significant fracture patterns in a multivariable setting. However, the sample size for this group of patients was too small to draw reliable conclusions based on logistic regression. We studied a specific setting (physician-staffed) and selected patients. External validity is therefore limited. Finally, even though PCCDs are considered to be cost-effective by some^21^, costs may be an important issue in low-income settings.

## Conclusions

In this large prehospital study, one out of six patients with a prehospital PCCD had a final diagnosis of a significant pelvic ring injury, whereas one out of four patients with a final diagnosis of a significant pelvic ring injury had no PCCD applied in the field. Further studies allowing for a better identification of patient-, physician- or accident-related factors associated with prehospital PCCD omission would be helpful to improve PCCD placement in the prehospital setting.

## Data Availability

The datasets used and/or analyzed during the current study are available from the corresponding author on reasonable request.
